# Social media reveal that charismatic species are not the main attractor of ecotourists to sub-Saharan protected areas

**DOI:** 10.1038/s41598-017-00858-6

**Published:** 2017-04-10

**Authors:** Anna Hausmann, Tuuli Toivonen, Vuokko Heikinheimo, Henrikki Tenkanen, Rob Slotow, Enrico Di Minin

**Affiliations:** 1 Amarula Elephant Research Programme, School of Life Sciences, University of KwaZulu-Natal, Durban, 4041 South Africa; 2 Department of Geosciences and Geography, University of Helsinki, FI-00014 Helsinki, Finland; 3 Department of Genetics, Evolution and Environment, University College, London, UK

## Abstract

Charismatic megafauna are arguably considered the primary attractor of ecotourists to sub-Saharan African protected areas. However, the lack of visitation data across the whole continent has thus far prevented the investigation of whether charismatic species are indeed a key attractor of ecotourists to protected areas. Social media data can now be used for this purpose. We mined data from Instagram, and used generalized linear models with site- and country-level deviations to explore which socio-economic, geographical and biological factors explain social media use in sub-Saharan African protected areas. We found that charismatic species richness did not explain social media usage. On the other hand, protected areas that were more accessible, had sparser vegetation, where human population density was higher, and that were located in wealthier countries, had higher social media use. Interestingly, protected areas with lower richness in non-charismatic species had more users. Overall, our results suggest that more factors than simply charismatic species might explain attractiveness of protected areas, and call for more in-depth content analysis of the posts. With African countries projected to develop further in the near-future, more social media data will become available, and could be used to inform protected area management and marketing.

## Introduction

Protected area management is crucial to enhance species persistence and reverse the biodiversity crisis^1^. Resources for protected area management are woefully inadequate^2^. Nature-based tourism can help generate funding needed to cover important management costs in protected areas^3–7^. Ecotourism, particularly, has been long promoted for its importance in supporting both biodiversity conservation and economic development^8, 9^. Sub-Saharan Africa is one of the top ecotourism destinations in the world^10^. Charismatic megafauna, such as the Big Five (lion - *Panthera leo*; leopard - *Panthera pardus*; elephant - *Loxodonta Africana*; buffalo - *Syncerus caffer*; black and white rhino - *Diceros bicornis* and *Ceratotherium simum*)^4, 11, 12^ or primates (gorilla spp. - *Gorilla gorilla* and *Gorilla beringei beringei*; chimpanzee - *Pan troglodytes*)^13^ are considered the main attractor of ecotourists to sub-Saharan African protected areas. Besides supporting management activities, funding from ecotourism can also help reduce important costs human communities pay for living alongside charismatic, yet dangerous, species^14^.

Besides the presence of charismatic megafauna, a wide range of other characteristics underpin nature-based tourism in protected areas^15, 16^. These include factors such as broader biodiversity (e.g. species richness^17^; threatened species and habitat types^18^; less charismatic biodiversity^11, 19^) and aesthetic of landscape (e.g. vegetation quality^20^). Geographical factors, such as accessibility (e.g. travel time^15^; trails and roads^21^), or degree of human influence (e.g. cultural landscapes^20^; overcrowding^19^) are also considered important. Furthermore, the socio-economic conditions of a country (e.g. political stability) also affect ecotourism visitation^22, 23^.

Thus far, studies assessing factors affecting tourists’ visitation patterns have focused on protected area visitation statistics^15, 23^. However, information about visitor numbers are generally costly (e.g. through survey-based methods) or difficult to collect (e.g. most parks are open access for recreation)^5^. Therefore, this information is often available only for few, well-known, protected areas. Alternatively, social media are increasingly being used as a cost-effective and rapid way to explore tourists’ visitation patterns^24–26^ or hotspots of human activities^27^. While data on visitor numbers can be scarce^5^, social media data are widespread and can, in some cases, be used as a proxy for tourism visitation rates^24, 27^. Therefore, data from social media can potentially be used as a new way to investigate which factors affect protected area attractiveness at continental or even global level. This is the challenge we addressed here.

**Figure 1:**
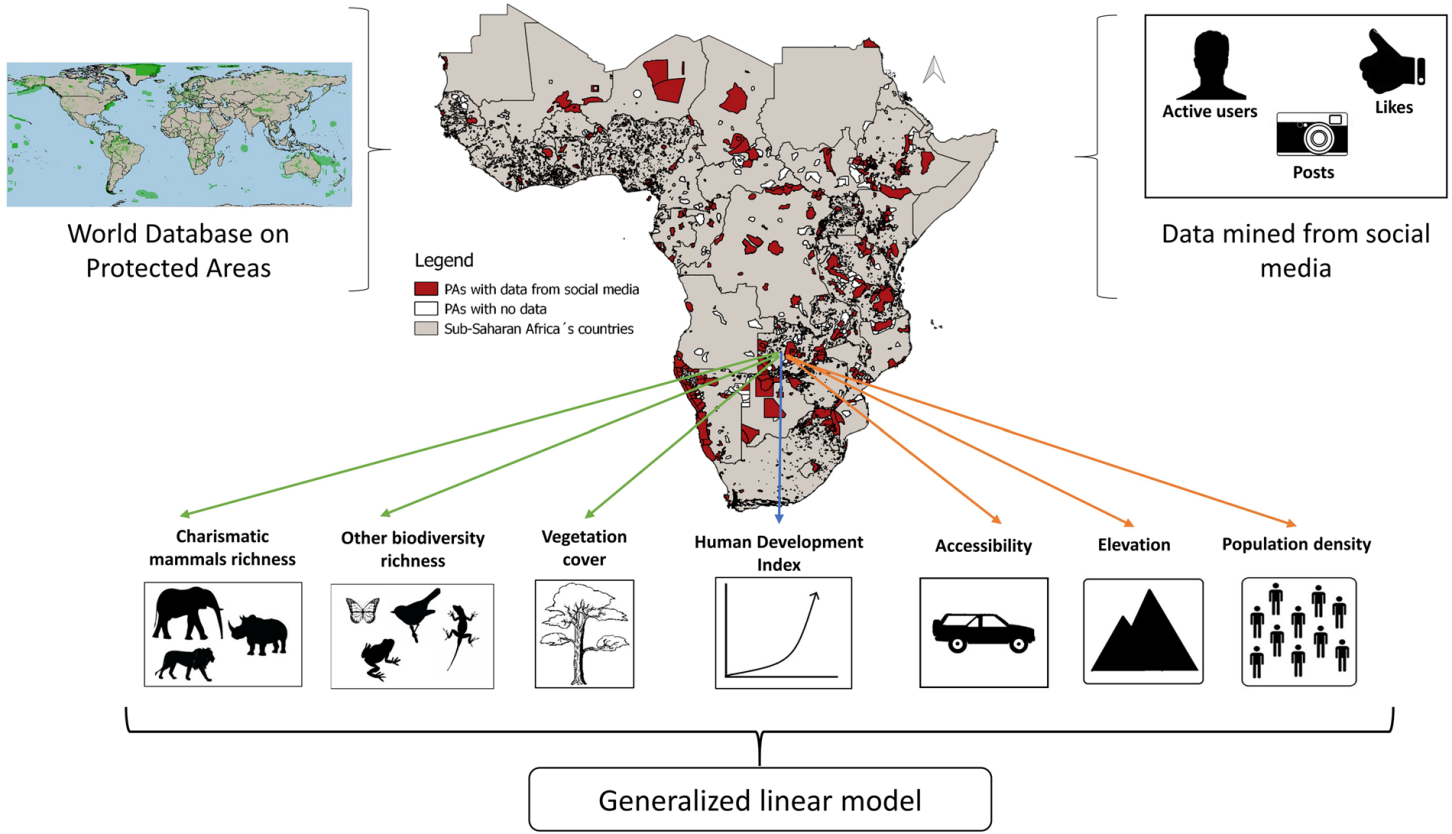
Logical framework of the study. For each protected area with data available from social media, biological (green arrows), geographical (orange arrows) and country level (blue arrow) attributes were also obtained and used in the generalized linear model as explanatory variables. Maps were created in QGIS 2.8.1 (URL http://www.qgis.org/en/site/). All images were generated by the authors.

In this study, we explored which socio-economic, geographical and biological factors explain social media use in sub-Saharan protected areas. Particularly, we were interested in understanding whether the number of charismatic species was an important contributor to social media usage in protected area. To do this, we used georeferenced Instagram pictures, posted within sub-Saharan African protected areas during 2015 to explore the effect of potential biological (i.e. richness of charismatic megafauna, richness of other biodiversity, vegetation cover), geographical (i.e. accessibility, elevation, population density) and socio-economic (i.e. Human Development Index [HDI]) factors on the density of active users, posts and likes (see framework in Fig. 1). In particular, we used generalized linear models with site- and country-level deviations to explore 1) which protected area and country level factors affect the use of social media; and 2) whether different explanatory variables explained the three response variables (i.e. the density of users, posts and likes). A total of 969 protected areas located in 41 countries (Table S1 in Appendix S1), for which social media data were available, were included in the analysis (Fig. 1). For almost half of the countries, we mined social media from protected areas covering ≥ 50% of the total area designated as protected (Figure S1 in Appendix S1). A total of 92,832 posts, posted by 55,756 active users, and liked 6,373,836 times were analyzed.

## Results

**Figure 2:**
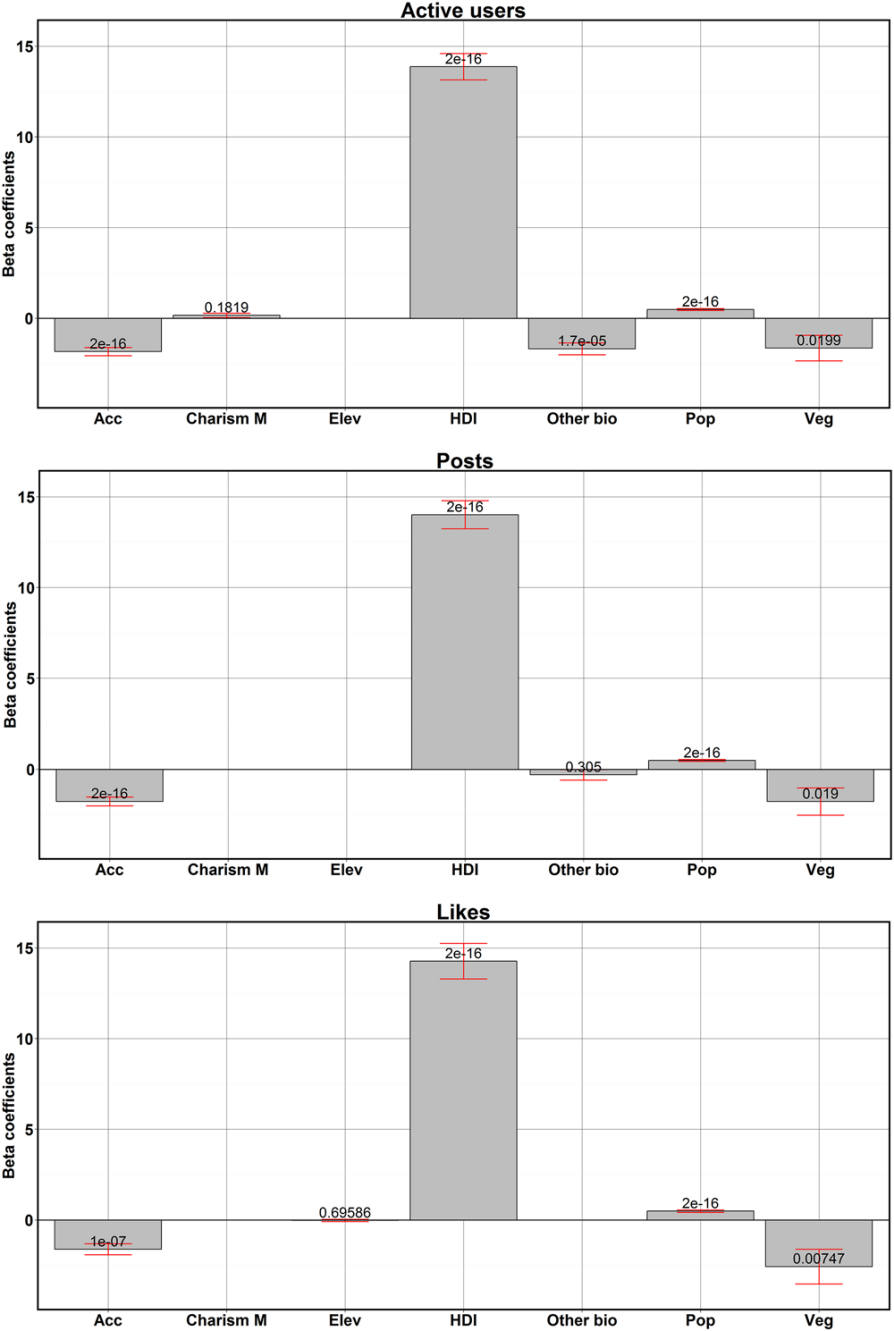
Beta coefficients of best predictors, averaged among the 6 top models of each response variable explaining use of social media in protected areas. The red bars show the confidence interval for each coefficient. The number over each bar are p-values and refer to the statistical significance. Figure S2 in Appendix S1 shows the values corresponding to this figure.

**Figure 3:**
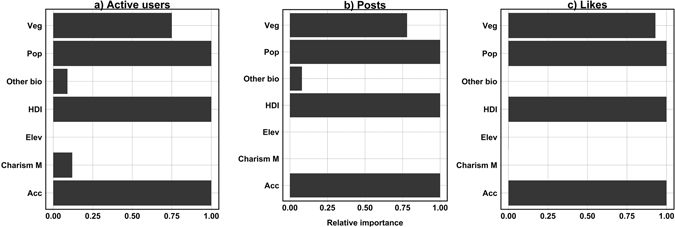
Overall weights of relative importance of 6 top predictors, averaged among top 6 models of each response variable.

The 6 top-ranked models, for each of the three response variables, describing the use of social media in protected areas are summarized in Table 1. The most important variables affecting social media usage across all models were HDI, accessibility, population density and the mean vegetation cover (Fig. 2). The country-level HDI was the strongest predictor, with coefficients up to four times higher than the other variables, across all models (Fig. 2). The positive sign of the coefficient indicates that social media usage was higher in protected areas in more developed countries. Accessibility was the second strongest variable predicting density of active users and posts. Specifically, accessibility had a negative sign, meaning that social media usage was higher in more accessible protected areas (Fig. 2). Moreover, population density was positively affecting the use of social media across all models (Fig. 2), meaning that social media use was higher in protected areas with higher density of people living around them. Vegetation cover had a negative sign, meaning that protected areas with more dense vegetation had lower social media use, and, in particular, pictures received fewer likes (Fig. 3). While less charismatic species (other biodiversity) was found to be less important compared to the other variables (Fig. 3), it was found to be statistically significant for number of active users in protected areas (Fig. 2). Specifically, less charismatic species (other biodiversity) had a negative sign, meaning that protected areas with higher species richness had lower densities of active users. The other variables considered in the models were less important (Fig. 3).

**Table 1:** Top-ranked predictors of social media usage within Sub-Saharan Africa protected areas.

Response variable	Model	No of variables	AIC weight	AIC	Delta	% of deviance explained
User	HDI + Acc + Pop + Veg	4	0.75	3230.610	0.000	54.00%
	HDI + Acc + Pop	3	0.130	3234.060	3.460	54.30%
	HDI + Acc + Cha M + Pop	4	0.120	3234.270	3.660	30.10%
	HDI + Acc + Oth bio + Pop	4	0.000	3234.620	4.010	54.10%
	Acc + Cha M + Pop	3	0.000	3572.400	341.790	30.70%
	Acc + Pop	2	0.000	3577.580	346.970	32.40%
Post	HDI + Acc + Pop + Veg	4	0.780	3343.280	0.000	50.60%
	HDI + Acc + Pop	3	0.130	3346.830	3.550	50.90%
	HDI + Acc + Oth bio + Pop	4	0.080	3347.760	4.490	27.60%
	Acc + Pop + Veg	3	0.000	3637.890	294.610	50.60%
	Acc + Oth bio + Pop	3	0.000	3638.680	295.400	29.70%
	Acc + Pop	2	0.000	3660.210	316.930	30.70%
Likes	HDI + Acc + Pop + Veg	4	0.930	3738.250	0.000	38.10%
	HDI + Acc + Pop	3	0.070	3743.440	5.190	36.50%
	HDI + Pop + Veg	3	0.000	3764.760	26.510	36.50%
	HDI + Elev + Pop + Veg	4	0.000	3766.610	28.350	38.70%
	Acc + Pop + Veg	3	0.000	3930.670	192.420	22.30%
	Acc + Pop	2	0.000	3963.090	224.830	19.40%

Akaike Information Criteria (AIC) weights represent the probability of the model being the best model.

For density of active users, the top-ranked model had an Akaike’s information criterion (AIC) weight of 0.75, explaining 54% of the deviance. For density of picture posted, the AIC weight was 0.78 and the deviance explained approximately 51%. For the density of likes the AIC weight was 0.93 but the deviance explained was 38%.

## Discussion

Overall, we found that richness of charismatic species had no influence on the use of social media in sub-Saharan Africa’s protected areas. This means that the number of highly iconic species which can be potentially found in a protected area, did not affect protected area visitors’ posting on social media. Interestingly, protected areas with higher richness of other species had fewer users posting on social media. Meanwhile, other factors, including both the socio-economic condition of countries and the geographical characteristics of the site, were more important in explaining the use of social media in sub-Saharan protected areas. In particular, protected areas located in more developed countries, which were more accessible and with more people living nearby, had higher densities of active users and posts on social media. Finally, protected areas with more open vegetation had higher densities of likes on social media.

While large-bodied mammals are considered the most important flagships for conservation in sub-Saharan Africa^11, 28^, we found that their presence did not affect the amount of active users, posts and likes on social media across sub-Saharan Africa’s protected areas. Besides charismatic wildlife-viewing, many tourists may also prefer visiting protected areas for their cultural, recreational value^29^, and visit places which allow for activities, such as hiking or biking, which are normally forbidden in parks where charismatic, dangerous animals are present^21^. Other studies show that, when looking at the content of pictures shared across different types of nature-based destinations, a variety of cultural uses, including recreation and aesthetic appreciation, are the most common subject among pictures^30^. In accordance, we found that areas with open vegetation (generally attractive to people as they allow views in the distance^31^), had higher use of social media, and received more likes, across different protected areas in sub-Saharan Africa. Viewsheds are key aspects of the visual landscape affecting visitor’s experiences^32^ and part of the sense of place sought by tourists in protected areas^33^. In addition, while in other regions (e.g. Finland^18^), or contexts (e.g. people expressing willingness to visit^17^), biodiversity appeared to underpin tourism attractiveness of protected areas, we found that areas with higher richness of species had fewer users active on social media. In our study area, higher species richness is found in moist tropical forests^34^ of central Africa’s countries, where protected areas receive fewer tourists due to less developed infrastructures (e.g. roads, cellphone coverage) and political or security issues^35^. Therefore, information about the use of social media in relation to the presence of species may be further explored in future studies. At the same time, content analysis of pictures may help reveal stronger relations between social media use and e.g. charismatic species.

The socio-economic condition of countries affects tourism patterns worldwide, with higher number of tourists visiting wealthier nations^15^. Similarly, we found that social media use in sub-Saharan Africa’s protected areas followed the same pattern, with more usage in countries with enhanced socio-economic conditions. Lack of provision of services and remoteness may discourage tourists’ visitation in the first place^36^. Moreover, gaps in mobile network coverage and the lack of smartphone devices may limit the geographical representativeness of social media data^37, 38^. As tourism expansion and economic growth of nations are interrelated^39^, social media potential will increase as many of these countries will also improve their development. Meanwhile, information obtained from more frequented sites, and from where data from social media is available, could be used as a first approximation for similar areas where data are scarce.

At a site level, our results show that variation of social media use across different protected areas well reflects tourist’s behavior in relation to geographical attractiveness of protected areas across sub-Saharan Africa. Similarly to previous studies, we found that better accessibility and higher density of people living nearby protected areas positively affect not only visitation rates^15, 25^, but also the use of social media. Highly populated areas around the borders of protected areas might also imply higher provision of tourists’ services and infrastructure^36^, including cellphone coverage^40^. On the other hand, such areas may be subjected to higher human pressure, such as environmental alteration, depletion of resources^41^, and threat to biodiversity, such as edge effect, especially in smaller areas^42^. Data from social media may be used to identify and monitor the use of sensitive locations by tourists, in order to inform conservation and management.

Different metrics of social media have been used to explore various aspects of tourists’ behavior, such as active users for assessing visitation^24^, amount of posts for exploring geographical hotspots of preferences^27, 43^ and likes to investigate engagement with specific subjects from the broader network^44, 45^. We found that all these metrics are affected by the same predictors is sub-Saharan Africa. However, vegetation openness was more important to receive more likes, while species richness was less important to explain higher densities of users. Deviance explained by our best models, especially for likes, suggests that other aspects not considered in this study may also influence the use of social media in protected areas. For example, individuals’ personalities and behavior on social media^46^, and the content of pictures^47^, may affect appreciation of pictures. Moreover, opportunities for biodiversity-related activities, such as hiking or camping, which were not considered in this study, might also be important aspects affecting social media usage, as they affect tourists’ decision-making^19^. However, posts on social media may not reveal the socio-economic background of different protected areas users. Future studies will require a more accurate differentiation between e.g. tourists, researchers, managers, and inhabitants. Future studies should also explore the profile of the social media users, e.g. by implementing deep learning algorithms, to overcome this limitation.

In conclusion, our results show that social media data can potentially be used as a first approximation to understand spatial preferences of tourists for nature-based experiences across protected areas in sub-Saharan Africa. Socio-economic development of countries and geographical characteristics of each site, and not the presence of species, were key aspects affecting visitation and attractiveness in sub-Saharan Africa’s protected areas. The potential of using social media data to inform conservation and ecotourism in sub-Saharan Africa will likely increase in the future, as some countries will improve their socio-economic conditions. Meanwhile, protected area managers and other conservation stakeholders in areas where social media are more commonly used, may take advantage of data uploaded by tourists to monitor the spatio-temporal variations of the use of cultural services, and inform conservation and ecotourism marketing. For example, social media data may help understand interests in biodiversity-related activities and be used to promote ecotourism in sites which lack charismatic species^19^. Content analysis of social media may help understand preferences for species^48^, and help identify more attractive protected areas in Africa, where ecotourism can be used as a tool to support conservation^49^. However, further analyses are needed in order to better understand the relationship between biodiversity and social media use in protected areas, including validating social media content with traditional surveys^48^, and exploring potential biases in the social media user population.

## Methods

### Study area and social media data

The framework of our study is presented in Fig. 1. We downloaded geo-referenced borders of sub-Saharan Africa’s protected areas from the World Database on Protected Areas (WDPA) (https://www.protectedplanet.net/ Accessed on June 2016). We considered all protected areas were data from social media was available (Fig. 1).

For each protected area, we collected geo-referenced pictures posted on Instagram within the border of the area (Fig. 1). Only sites over 10 square kilometers^15^ were considered in order to avoid biases related to social media location inaccuracy^37^. Data were accessed through the application programming interface (API) (https://www.instagram.com/developer/) available from the platform. Geo-referenced posts were sampled each first week of every month of the year 2015. We collected three metrics of social media usage, i.e. total number of active users (users who had posted at least 1 picture per day is counted once per day), posts (pictures), and likes of pictures posted in the area.

### Potential predictors of social media use in protected areas

We selected 8 variables that are considered to affect tourists’ preferences for nature-based tourism in protected areas, and which could potentially be related to social media use (Table 2). These variables were site specific, i.e. biological and geographical, or country-specific, i.e. socio-economic (Fig. 1). All mapping was performed in QGIS version 2.8.1.

**Table 2:** Potential predictors used in the GLM to explain social media use by tourists visiting sub-Saharan Africa’s PAs.

Predictor	Variable	Data type	Data origin	Source
Biological	Richness of charismatic mammal species	Count data	IUCN Red list database.	http://www.iucnredlist.org/
	Richness of less charismatic mammal species	Count data	IUCN Red list database.	http://www.iucnredlist.org/
	Richness of other less charismatic species	Count data	IUCN Red list database.	http://www.iucnredlist.org/
	Vegetation cover	EVI (MOD13A3), raster, continuous	Land Processes Distributed Active Archive Center (LP DAAC) managed by the NASA Earth Science Data and Information System (ESDIS) project.	http://modis.gsfc.nasa.gov/data/dataprod/mod13.php
Geographical	Accessibility	Raster, continuous	Global Environment Monitoring Unit - Joint Research Centre of the European Commission, Ispra Italy.	http://maps.tnc.org/gis_data.html
	Elevation	Raster, continuous	ASTER GDEM from NASA and METI	http://asterweb.jpl.nasa.gov/gdem.asp
	Population density	Raster, continuous	Global Rural-Urban Mapping Project, Socioeconomic Data and Applications Center (sedac).	http://sedac.ciesin.columbia.edu/data/set/grump-v1-population-density
Socio-economic	Human Development Index (HDI)	Continuous	Human development reports of the United Nations Human Development Programme	http://hdr.undp.org/en/content/human-development-index-hdi

Biological factors were considered in order to assess whether biodiversity or landscape-related variables affected the use of social media in protected areas (Fig. 1). Biodiversity variables were obtained by calculating richness (sum of species occurring in the area) of 9,916 species of vertebrates, invertebrates and plants, occurring in sub-Saharan Africa, for each site. Species occurrence maps were obtained from the IUCN Red list database (Accessed in May 2015), which is the latest updated source of information about species ranges that is also publicly available. However, range maps overestimate the true area occupied by species, as it may include areas where species presence is probable but not confirmed^50^. Charismatic megafauna, which are particularly attractive to tourists in sub-Saharan Africa^11^, were considered separately from other less charismatic species in order to explore whether the use of social media among tourists was affected by the richness of these species in protected areas. Charismatic mammals included 40 large-bodied mammal species, with average body weight larger than 100 kg^51, 52^. As other less charismatic biodiversity may also be attractive for different markets of tourists^19^, we grouped richness of amphibians (999 species), arthropods (750 species), birds (2246 species), reptiles (723 species), plants (603 species) and freshwater fish (3350 species), and mammal species (1245 species) with average body weight smaller than 100 kg together as “other biodiversity”. Moreover, we focused our analysis only on continental Africa, excluding Madagascar and other islands, as we wanted to assess the importance of large-bodied mammals.

Vegetation cover was considered as another biological factor as a proxy for landscape aesthetic. Open vegetation is a key aesthetic attractor for landscape preferences^53^, affecting tourists’ decision-making for nature-based experiences in protected areas^19^. We used MODIS Enhanced Vegetation Index (EVI) as a measure for vegetation cover (Table 2) in order to explore whether more open vegetation would also affect the use of social media in protected areas. EVI is optimized for characterizing vegetation state in areas with dense canopy. Data were downloaded for the period of February 2000 to December 2014 at 1 km resolution at the equator.

Geographical variables included accessibility, elevation and population density as potential predictors of social media use in protected areas. More accessible areas receive more tourists than remote ones^15^. In order to explore whether higher accessibility is also driving the use of social media in protected areas, we calculated mean accessibility values of a 10 km buffer zone, built around each protected area. Values were extracted from a global map of accessibility (Nelson 2008), developed by the European Commission and the World Bank (Table 2). Accessibility values represent the travel time, by land or water, from the nearest major city to each protected area (cities with 50,000 or more people in the year 2000)^54^. Units of time represents the “cost” of travelling where higher values are more costly and smaller values less costly, thus indicating better accessibility.

Elevation was considered as another geographical attribute, as tourists’ preferences for nature-based destinations may also be influenced by topography. For example, elevation (e.g. costal or mountain areas^55^) and slope (e.g. hiking opportunities^56^), may affect aesthetic of landscapes and preferences for nature-based experiences in protected areas. In order to determine whether altitude of protected areas may also affect the use of social media we used data from Aster Global Digital Elevation Model v002 (ASTG TM) at 30 m resolution to extract mean elevation values of each site (Table 2).

Moreover, density of population living nearby was also considered among the geographical variables, as tourists visitation rates is positively affected by population density^15, 57^. More populated areas are more likely to provide infrastructures, such as mobile phone coverage^40^, which can affect spatial patterns of social media use. In order to understand whether population density outside protected areas may also affect social media usage inside the area, we estimated mean population densities around a 10 km buffer zone built around each area. Population density values were extracted from the Global Rural-Urban Mapping Project, Version 1 (GRUMPv1) (http://sedac.ciesin.columbia.edu/data/set/grump-v1-population-density) and estimated at 10 km resolution at the equator (Table 2).

Finally, we considered the socio-economic condition of countries as potential predictor of social media use in protected areas. Gross domestic product of countries affects tourists visitation rates^23^, with fewer visitors in poorer and politically instable areas. While tourism is increasing worldwide, international tourism in Africa has decreased by 3% in 2015, due to slow economic growth and struggles related to health and security^35^. However, trends change among sub-Saharan Africa countries. In order to explore whether the different socio-economic conditions of countries would affect social media use in protected areas, we considered the HDI, developed by UNEP (Table 2). The HDI is the result of a geometric mean between three indexes of human development, i.e. life expectancy, education and gross national income per capita, in each country. The index was chosen in this study as it summarizes information about countries’ socio-economic condition, and represents an official indicator based on data sources provided by major statistical agencies of the United Nations.

### Statistical analysis

We used an information theoretic approach^58^ and a generalized linear mixed effect model (GLMM) to explain the use of social media in sub-Saharan Africa’s protected areas. Three response variables, representing different metrics of use of social media, were used, namely density of social media active users, density of posts and density of likes in each protected area. Densities were calculated as number of active users, posts or likes per km^2^ of the area were they occurred. The GLMM accounted for both fixed and random effects. Biological (richness of charismatic and other biodiversity, and vegetation cover), geographical (accessibility, elevation and population density) and socio-economic (country-related human development index) were used as fixed effect in all the models. Due to high heterogeneity in the spatial distributions, between countries (Table S2 in Appendix S1) and protected areas (Figure S2 in Appendix S1), of our response variables, two levels, i.e. site (protected area) and country of each site, were used as random effects in the models. This is, in order to allow our models to account for spatial variability, by including regression coefficients which are constant across sites and countries. To fit our model, we used a binomial family type with logit-link distribution of errors. As values of the variables had skewness of distributions, all explanatory variables (charismatic megafauna richness, other biodiversity richness, vegetation cover, accessibility of the buffer area, elevation, population density of the buffer area and HDI of country), except vegetation cover (values range between 0 and 1) were log-transformed. We used the *Corrgram* package in R^59^, with a cut-off of r = 0.80, to test for correlation among explanatory variables. We only selected variables with the strongest effect on social media usage which were not correlated in order to avoid multicollinearity among variables. Next, we implemented multimodel averaging in the R version 3.0.2^60^ package *glmulti* ^61^. Multimodel averaging^58^ is commonly used in ecology and conservation science to rank, based on the Akaike Information Criteria, all possible fitted models from best to worse and then averaging the coefficients values across models to reduce uncertainty. In addition, we measured the relative importance of the most important predictor variables^62^ by using Akaike weights over the six top-ranked models and a cut-off of w = 0.80. Percentage of deviance explained by each model was used as a measure of goodness of fit.

## Electronic supplementary material

Appendix S1
