# Supplementary material for: Social media reveal that charismatic species are not the main attractor of ecotourists to sub-Saharan protected areas
Source: Sci Rep. 2017 Apr 10;7:763. doi: 10.1038/s41598-017-00858-6 (PMC5429685; doi:10.1038/s41598-017-00858-6)
Supplement: Supplementary file 1 — Appendix S1 [file 41598_2017_858_MOESM1_ESM.pdf]

Social media reveal that charismatic species are not the main attractor of ecotourists to sub-Saharan protected areas.

Anna Hausmann[1]; Tuuli Toivonen[2]; Vuokko Heikinheimo[2]; Henrikki Tenkanen[2]; Rob Slotow[1,3]; Enrico Di Minin[1,2]

[1]Amarula Elephant Research Programme, School of Life Sciences, University of KwaZulu-Natal, Durban 4041, South Africa;

[2] Department of Geosciences and Geography, University of Helsinki, FI-00014, Helsinki, Finland

[3]Department of Genetics, Evolution and Environment, University College, London, United Kingdom

Contact: Anna Hausmann, +358 417088225; email: anna.hausmann87@gmail.com

Supplementary Appendix S1

Tables

Table S1. Total number of protected areas, areas covered by social media, sum of posts, active users and likes in each sub-Saharan Africa's country.

Country code	Country name	Protected areas	Covered by social media	Posts	Active users	Likes
ZAF	South Africa	1012	310	61816	36794	3598295
KEN	Kenya	245	105	9178	5567	843701
TZA	Tanzania	603	71	8194	4864	935169
NAM	Namibia	146	64	2874	1882	268929
BWA	Botswana	18	12	2335	1350	225235
UGA	Uganda	703	43	2055	1276	98111
ZMB	Zambia	969	38	770	539	71033
NGA	Nigeria	518	57	797	513	26901
ZWE	Zimbabwe	174	30	564	355	93933
COD	Democratic Republic of the Congo	45	28	499	343	34376
SEN	Senegal	115	12	500	269	19933
MOZ	Mozambique	46	14	480	246	14942
ETH	Ethiopia	95	32	322	201	18798
GNQ	Equatorial Guinea	13	3	357	199	15117
TCD	Chad	14	8	247	174	20318
GHA	Ghana	257	19	252	155	14908
RWA	Rwanda	6	4	243	133	12849
CIV	Ivory Coast	240	12	159	124	6440
SSD	South Sudan	24	3	159	117	2614
AGO	Angola	12	4	140	99	5245
GAB	Gabon	26	13	202	96	9937
CMR	Cameroon	97	6	113	95	9043
LSO	Lesotho	2	2	156	84	4177
SWZ	Swaziland	20	9	136	74	3391
MWI	Malawi	56	14	63	52	2355
SLE	Sierra Leone	36	5	37	30	2171
GMB	Gambia	12	4	25	21	934
LBR	Liberia	16	3	27	19	1008
GNB	Guinea-Bissau	23	8	35	16	11430
BFA	Burkina Faso	75	7	21	15	692
MLI	Mali	26	4	15	14	526
MRT	Mauritania	4	4	24	11	184
COG	Republic of Congo	19	4	7	6	414
BEN	Benin	28	4	5	5	220
GIN	Guinea	104	2	5	5	106
TGO	Togo	60	2	9	4	208
CAF	Central African Republic	25	4	6	4	56
NER	Niger	15	4	4	4	136
ERI	Eritrea	4	1	1	1	1
BDI	Burundi	7	0	0	0	0
SDN	Sudan	1	0	0	0	0

Table S2. Beta coefficient values, standard errors and statistical significance at $p<0.05$ of best predictors averaged among the 6 top models of each response variable explaining use of social media in protected areas. Visualization is provided in Figure 2 in the main text.

Response variable	Parameter	Coefficient	Standard Errors	p-value
Active users	Veg	-1.64281	0.70539	0.0199
	Acc	-1.83418	0.22423	2.00E-16
	Pop	0.48319	0.04991	2.00E-16
	HDI	13.88167	0.72318	2.00E-16
	Charism M	0.16186	0.12125	0.1819
	Other bio	-0.32766	0.27410	0.2319
	Elev	-	-	-
Posts	Veg	-1.77375	0.75524	0.019
	Acc	-1.76793	0.23899	2.00E-16
	Pop	0.49152	0.05264	2.00E-16
	HDI	14.00968	0.77376	2.00E-16
	Other bio	-0.30117	0.29356	0.305
	Charism M	-	-	-
	Elev	-	-	-
Likes	Veg	-2.56672	0.95951	0.00747
	Acc	-1.61227	0.30125	1.00E-07
	Pop	0.48909	0.06548	2.00E-16
	HDI	14.2693	0.97738	2.00E-16
	Elev	-0.02387	0.06107	0.69586
	Charism M	-	-	-
	Other bio	-	-	-

Table S3 – Average dimension (km^2), densities of social media active users, posts and likes/km2 of country's protected areas. See Table S1 for definition of country codes. Countries are ordered according to average density of users.

Country code	Average area (km^2) protected in each site per country	Average of active users density	Average of posts density	Average of likes density
ZAF	657.361	0.605	0.855	51.844
UGA	783.429	0.358	0.538	32.206
BWA	15612.583	0.340	0.532	36.827
NGA	780.249	0.328	0.506	22.242
KEN	682.915	0.269	1.315	91.337
SWZ	109.538	0.252	0.385	9.051
SEN	2735.618	0.237	0.407	13.190
SSD	11781.400	0.205	0.277	4.313
GMB	51.700	0.155	0.193	6.170
ZMB	3412.569	0.115	0.152	19.643
CIV	3813.317	0.082	0.107	6.542
TZA	3966.554	0.065	0.118	6.548
GHA	686.524	0.061	0.073	3.118
NAM	4571.847	0.060	0.103	8.136
RWA	663.825	0.054	0.103	4.513
ZWE	1580.043	0.048	0.069	3.408
COD	9303.636	0.040	0.062	1.474
GNQ	1139.333	0.037	0.057	2.481
SLE	222.260	0.034	0.041	2.520
MWI	1012.585	0.033	0.038	1.374
LSO	3196.750	0.021	0.027	0.530
LBR	909.867	0.020	0.027	1.023
CMR	796.017	0.013	0.016	0.996
MOZ	5830.664	0.012	0.023	0.819
TGO	109.600	0.009	0.009	0.520
COG	3096.900	0.006	0.006	0.267
BFA	3866.600	0.006	0.013	0.289
GAB	2652.569	0.005	0.009	0.521
ETH	5451.003	0.005	0.007	0.338
AGO	9894.300	0.004	0.006	0.162
GIN	321.200	0.003	0.003	0.103
GNB	885.275	0.002	0.004	3.329
TCD	17965.325	0.002	0.002	0.160
ERI	622.700	0.002	0.002	0.002
MRT	4315.650	0.001	0.002	0.018
MLI	13032.025	0.001	0.001	0.036
BEN	2747.275	0.001	0.001	0.015
CAF	12928.450	0.000	0.000	0.002
NER	61214.475	0.000	0.000	0.003

Figure

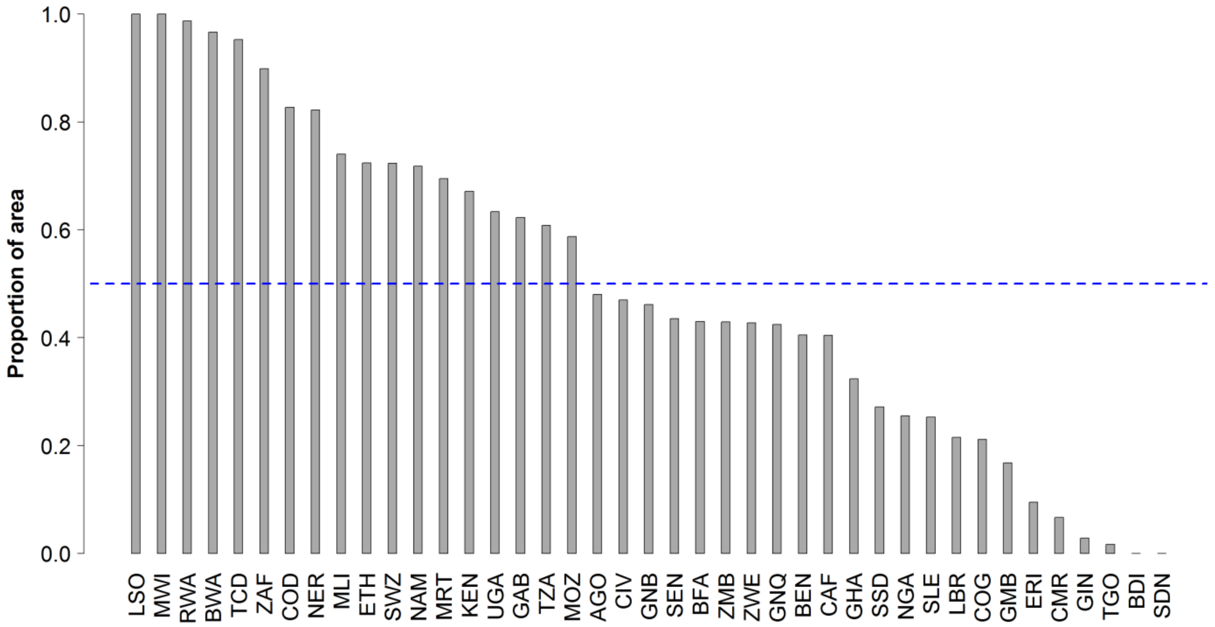

Figure S1. Proportion of total protected area coverage by social media data in each country of sub-Saharan Africa. Countries over the blue dotted line have more than 50% of protected area covered by social media.

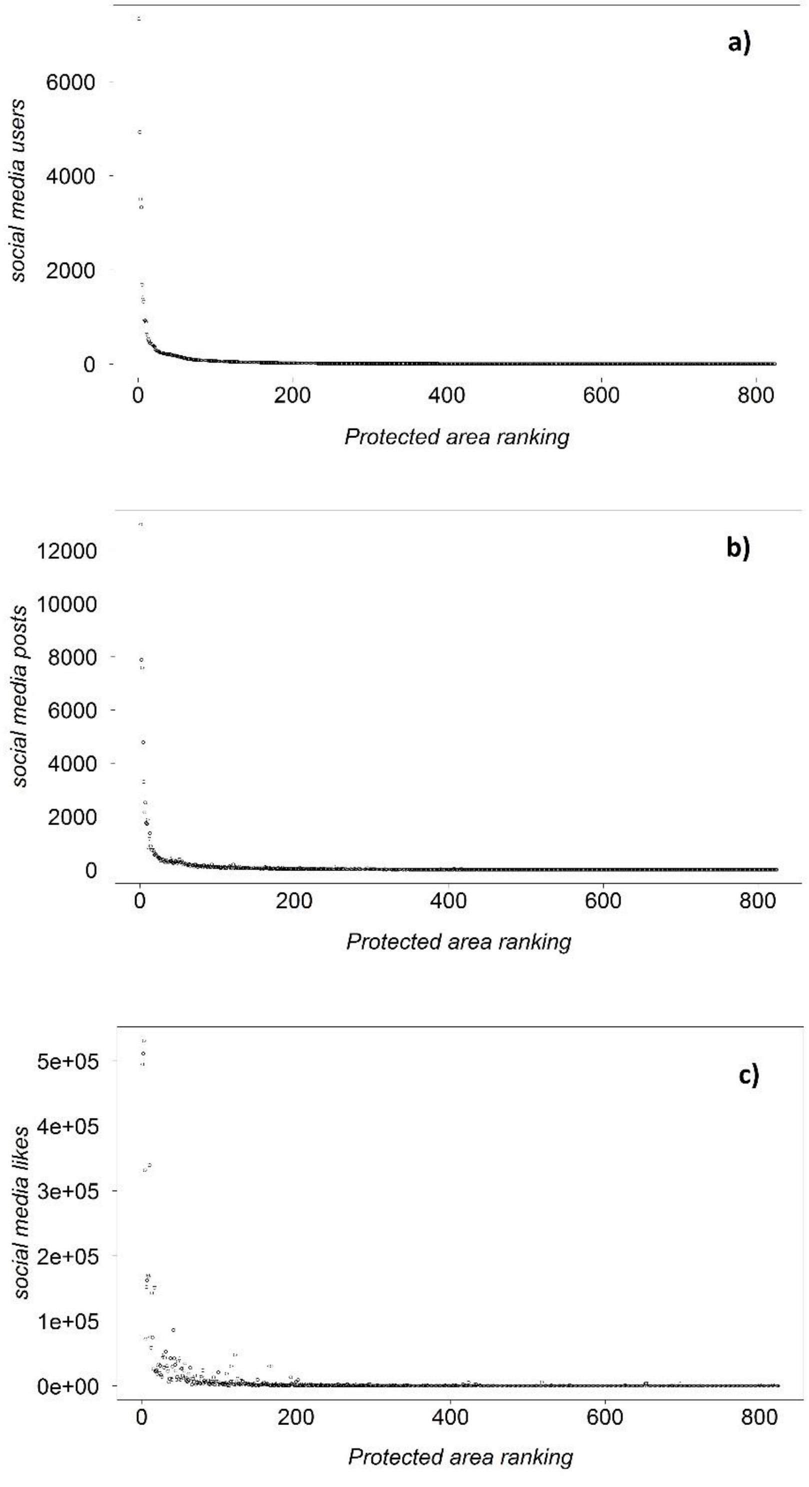

Figure S2 – Distribution of total number of active user (a), posts (b) and likes (c) collected for each sub-Saharan Africa's protected area, showed in a decreasing ranking according to number of active users.
